# Natriuretic peptides and fat metabolism

**DOI:** 10.1186/2050-6511-14-S1-O24

**Published:** 2013-08-29

**Authors:** Sheila Collins

**Affiliations:** 1 Diabetes and Obesity Research Center, Sanford-Burnham Medical Research Institute, Orlando, FL, USA

## 

The regulation of adipose tissue metabolism has for many years been the domain of insulin action to promote glucose uptake and lipogenesis, and the catecholamines of the sympathetic nervous system working through β-adrenergic receptors (βARs) and PKA to stimulate lipolysis and the release of these stored fatty acids. Catecholamines can also increase the machinery for uncoupled respiration in brown adipocytes - including the “browning” of cells in white adipose depots - together resulting in net energy expenditure. However we now know that this is not the whole story. Several years ago the receptors for the cardiac natriuretic peptides ANP and BNP were found to be expressed in adipose tissue. It was then noted that ANP could increase lipolysis via PKG phosphorylation of the same targets as PKA. Using cultured adipocytes, as well as mice with targeted deletions of GC-A/NPRA and NPRC, we have found that ANP/BNP can stimulate “browning” of adipocytes similar to that elicited by catecholamines. In earlier work, we established that βARs drive the transcription of brown adipocyte genes and respiratory uncoupling through a signaling cascade from PKA to p38 MAP kinase. The natriuretic peptides and PKG also utilize this novel p38 MAPK pathway such that the βARs and NPRA work together, additively increasing expression of brown adipocyte marker genes, as well as reflexively controlling each other’s components. These findings shed new light on the control of body fat and energy expenditure, with important implications for understanding obesity as well as cardiovascular disease such as hypertension and heart failure. Since obesity in humans as well as rodents is accompanied by increased levels of NPRC in adipose tissue, thus impairing signaling, and obese individuals often exhibit high blood pressure, therapeutic approaches that target the NP receptors may have the potential to increase energy expenditure, promote weight loss and reduce blood pressure.

